# Time on Therapy of Automated Peritoneal Dialysis with and without Remote Patient Monitoring: A Cohort Study

**DOI:** 10.1155/2022/8646775

**Published:** 2022-08-22

**Authors:** Mauricio Sanabria, Jasmin Vesga, Bengt Lindholm, Angela Rivera, Peter Rutherford

**Affiliations:** ^1^Baxter Renal Care Services-Latin America, Bogotá, DC, Colombia; ^2^Baxter Renal Care Services Colombia, Bucaramanga, Colombia; ^3^Renal Medicine and Baxter Novum, Karolinska Institutet, Stockholm, Sweden; ^4^Baxter Healthcare Corporation, Deerfield, Illinois, USA; ^5^Baxter Healthcare Corporation, Zurich, Switzerland

## Abstract

**Background:**

Remote patient monitoring (RPM) of patients undergoing automated peritoneal dialysis (APD-RPM) may potentially enhance time on therapy due to possible improvements in technique and patient survival.

**Objective:**

To evaluate the effect of APD-RPM as compared to APD without RPM on time on therapy.

**Methods:**

Adult incident APD patients undergo APD for 90 days or more in the Baxter Renal Care Services (BRCS) Colombia network between January 1, 2017, and June 30, 2019, with the study follow-up ending June 30, 2021. The exposure variable was APD-RPM vs. APD-without RPM. The outcomes of time on therapy and mortality rate over two years of follow-up were estimated in the full sample and in a matched population according to the exposure variable. A propensity score matching (PSM) 1:1 without replacement utilizing the nearest neighbor within caliper (0.035) was used and created a pseudopopulation in which the baseline covariates were well balanced. Fine & Gray multivariate analysis was performed to assess the effect of demographic, clinical, and laboratory variables on the risk of death, adjusting for the competing risks of technique failure and kidney transplantation.

**Results:**

In the matched sample, the time on APD therapy was significantly longer in the RPM group than in the non-RPM group, 18.95 vs. 15.75 months, *p* < 0.001. The mortality rate did not differ between the two groups: 0.10 events per patient-year in the RPM group and 0.12 in the non-RPM group, *p*=0.325.

**Conclusion:**

Over two years of follow-up, the use of RPM vs. no RPM in APD patients was associated with a significant increase in time on therapy, by 3.2 months. This result indicates that RPM-supported APD therapy may improve the clinical effectiveness and the overall quality of APD.

## 1. Introduction

Patient survival and length of treatment time on dialysis therapies are core indicators of the effectiveness of kidney replacement therapies [1, 2]. Peritoneal dialysis (PD) programs are characterized by a higher rate of technique failure than in-center hemodialysis (HD) programs, which reduces the time that the patient is able to remain on PD [3]. The peritoneal dialysis (PD) scientific community has made an effort to unify the various definitions of technique failure in PD [4, 5]. Also, in the age of person-centered care, it is essential to quantify the time that elapses in patients until an event occurs (for example, death or technique failure) to understand in an easy way what happens in the life cycle of the patient within PD therapy [6, 7]. The Standardized Outcomes in Nephrology–Peritoneal Dialysis (SONG-PD) initiative identified the most important PD outcomes and implemented standardized patient-reported outcomes [1]. This patient-centric initiative highlights the need to seek simplicity with binary outcomes and uses positive terminology. According to these recommendations, quantifying the time that patients stay on PD therapy (time on therapy) can be a straightforward and understandable way to present outcomes with a more positive connotation than presenting the binary outcome of death or using potentially pejorative terms such as “technique failure.”

In addition, the last five years have seen the introduction and wider use of remote monitoring programs for patients on automated peritoneal dialysis (APD) that may potentially influence time on therapy [8–13]. As reported by Wallace et al. [8], remote monitoring programs for APD patients offer the opportunity to better understand how therapy is being carried out in the patient's own environment, thus achieving greater confidence in the treatment and the potential for better clinical results. Regarding the specificities of the remote monitoring program, Sanabria et al. [9] have reported the way in which clinical teams can monitor whether the patient underwent treatment, if there were losses in treatment time or ineffective dialysis time, in addition to losses of treatment volume, anticipated drainage of the peritoneal cavity, and ultrafiltration profiles, all of the above added to the possibility of monitoring the patient's body weight or blood pressure. These programs may improve the effectiveness and outcomes of APD, and their usefulness has received increasing attention during the COVID-19 crisis [14, 15]. Specifically, the use of RPM in APD has reduced the frequency of hospitalization events and hospital days [11] and the frequency of PD technique failure compared to APD programs without remote monitoring [16].

This study aims to evaluate the effect of RPM in APD in terms of time on PD therapy compared to APD without RPM.

## 2. Materials and Methods

### 2.1. Study Design and Patients

This retrospective, observational, multicenter, cohort study of incident patients undergoing APD (defined as those receiving APD for more than 90 days) at the Baxter Renal Care Services network in Colombia was conducted between January 1, 2017, and June 30, 2019, with the end of follow-up within the study on June 30, 2021. Inclusion criteria were age above 18 years, diagnosis of kidney failure, and undergoing treatment by APD for 90 days or more. Exclusion criteria comprised pregnancy, those undergoing dialysis therapies due to nonkidney indications of dialysis such as congestive heart failure and liver cirrhosis, and those treated for less than one-month using APD-RPM. The patients were divided into two cohorts according to the use of RPM, which constitutes the exposure variable: (1) APD-RPM cohort: patients using the Homechoice Claria® device with the Sharesource® connectivity platform (Baxter Healthcare, Deerfield, USA) and (2) APD-without RPM cohort: patients using APD Homechoice® without RPM. Censored events were loss to follow-up, change of dialysis provider, change of APD modality (patients who started in the APD without RPM cohort and then switched to APD with RPM), and recovery of kidney function. Follow-up within the study was up to two years.

An intention-to-treat approach was used according to whether the patient used a remote monitoring program at the time of the inception of the cohort (APD-RPM) or not (APD-without RPM).

The study protocol was approved by the clinical research ethics committee of Renal Therapy Services Colombia (October 3, 2017, Minute, item number 0009-2017), which exempted informed consent as this study does not contain identifiable information and is a retrospective observational study.

### 2.2. Baseline Patient Characteristics

Demographic and clinical baseline variables included age, sex, race, diabetes history, socioeconomic level, school level, Charlson Comorbidity Index, center size, hemoglobin, phosphorus, potassium, albumin, and urine output in mL/day.

The exposure variable of interest was APD-RPM vs. APD-without RPM. The APD-RPM cohort was composed of patients using the Homechoice Claria® device with the Sharesource® connectivity platform (Baxter Healthcare, Deerfield, USA) and the APD without RPM cohort of patients using APD Homechoice® without the connectivity platform. Access to one or the other of the two cohorts depended solely and exclusively on the availability of each of the technologies in renal clinics and the patient's desire.

All data were obtained from the electronic medical records system (Versia®) and exported to an Excel® database. Data quality auditors carried out a data quality assurance process, focused on data integrity and dates, with emphasis on study outcomes. We did not find any missing data, so no data imputation was required.

### 2.3. Study Outcomes

The primary outcome was time on APD therapy, and the secondary outcome was the mortality rate over a two-year period follow-up. Censored events included a loss to follow-up, change of dialysis provider, change of APD modality, and recovery of kidney function. The study's follow-up period lasted up to two years.

### 2.4. Statistical Analysis

Descriptive statistics were used to report population characteristics, such as mean and standard deviation (SD) for normally distributed variables and median and interquartile range (IQR) for nonnormally distributed variables. In addition, all categorical variables were compared between HD groups using Pearson's *χ*^2^ test, and continuous variables were analysed with Student's *t*-test. Baseline differences between the groups were compared using standardized differences (where a value >10% was considered clinically meaningful). No data imputation procedure was performed to handle missing data. A propensity score matching (PSM) 1 : 1 without replacement utilizing the nearest neighbor within caliper (0.035) was used and created a subpopulation in which the baseline covariates were well balanced. The propensity score for each subject was calculated from a logistic regression model that included variables as predictors of the exposure status such as age, sex, black race, diabetes history, socioeconomic level, school level, Charlson Comorbidity Index, center size, hemoglobin, phosphorus, potassium, albumin, urine output in mL/day, and censored events. Fine & Gray multivariate analysis was performed to assess the effect of demographic, clinical, and laboratory variables on the risk of death, adjusting for competing risk events such as technique failure and kidney transplantation. In addition, we estimated the cumulative incidence of deaths by adjusting for competing risk events, and Pepe and Mori's statistical test was used to compare the equality of the cumulative incidence functions (CIFs) by exposure status. Also, we approached with Inverse Probability of Treatment Weighting (IPTW) using a propensity score to control differences between the groups and perform a sensitivity analysis for the direction of the observed effect. In statistical analyses, Stata 16® (StataCorp. 2019. Stata Statistical Software: Release 16. College Station, TX: StataCorp LLC.) was used.

## 3. Results

### 3.1. Patients

**Figure 1:**
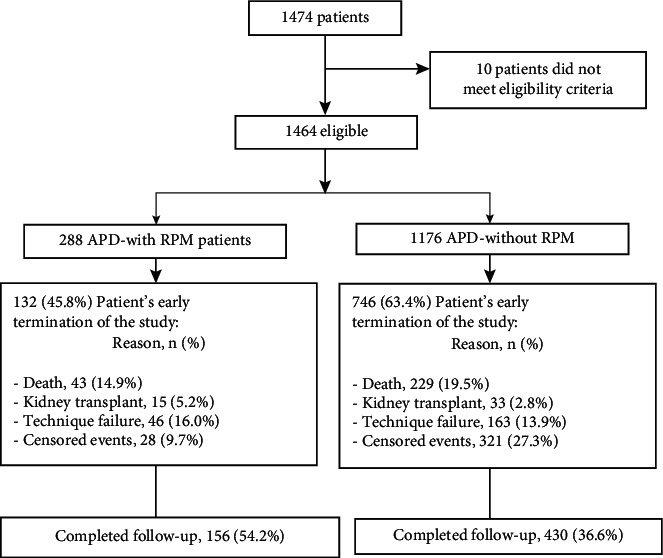
Flow chart of patients in the study.

A total of 1464 patients were included in the study, 288 in the APD-RPM group and 1176 in the APD-without RPM group (Figure 1); 586 completed the total follow-up time, 156 in the APD-RPM group and 430 in the APD-without RPM. The mean age of patients in the APD-RPM group was 60.2 ± 16.5 years, and 63.5% were men; in the APD-without RPM group, the mean age was 60.4 ± 16.1 years, and 58.9% were men. The proportion of patients with diabetes mellitus was 49.3% for APD-RPM vs. 53.5% for APD-without RPM, *p*=0.203. Baseline hemoglobin, albumin, potassium, phosphorus, and urine output ml/day were similar in the two groups; more details of the socio-demographic and clinical characteristics of the two populations are presented in Table 1.

### 3.2. Outcomes in the Full Sample

**Figure 2:**
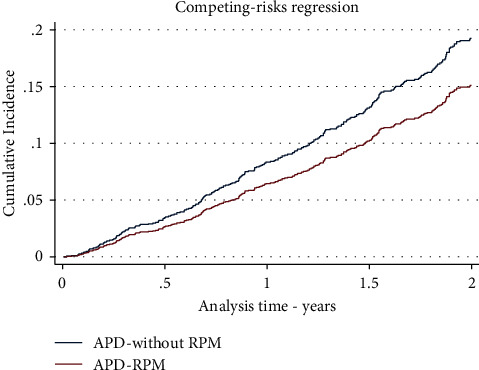
Mortality cumulative incidence function with competing events in the full sample. APD: automated peritoneal dialysis; RPM: remote patient monitoring.

There were 272 death events with an overall rate of 0.13 events per patient-year (95% CI: 0.12–0.15). The mortality incidence funtion adjusting for competing risks was for the APD-RPM group in the first year of 7.1% (95% CI: 4.5% to 10.6%), and at the second year of 15.9% (95% CI: 11.8% to 20.6%). For the APD-without RPM group, it was for the first year of 11.0% (95% CI: 9.2% to 12.9%), and at the second year of 23.6% (95% CI:21.0 % to 26.4%). See Table 2 and Figure 2. We compared the cumulative incidence function according to exposure status and observed a statistically significant difference when adjusting for technique failure (*p*=0.013) or kidney transplant (*p* = 0.014).

### 3.3. Matched Analysis

**Figure 3:**
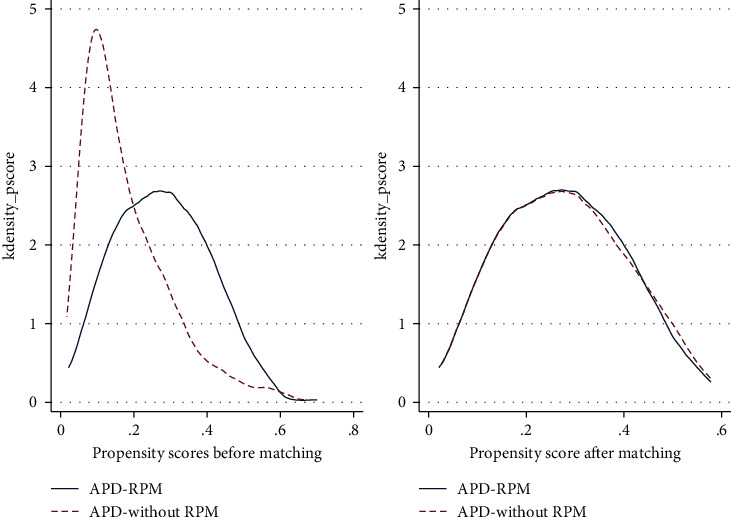
Distribution of propensity scores for the two cohorts before and after matching. APD: automated peritoneal dialysis; RPM: remote patient monitoring.

We reach an adequate balance between the two populations with the process of matching using propensity scores, see Figure 3. Statistically significant differences were observed in time on therapy when comparing APD-RPM with the APD-without RPM group, 18.95 (SD: 7.3) versus 15.75 (SD: 8.1) months *p* < 0.001. The time on therapy was greater for those with the RPM program by 3.2 months (95% CI: 1.93 to 4.46). The APD-RPM group was associated with a lower mortality rate per patient-year, although this difference was not statistically significant, 0.10 (95% CI: 0.07 to 0.13) versus 0.12 (95% CI: 0.09 to 0.16) events/person-year, *p*=0.325 (Table 3).

### 3.4. Weighted Analysis

After the process of cohort's weighting using IPW, the APD-RPM group was associated with a significant increase of time in therapy, by 2.5 months, 95% CI (1.16 to 3.85); *p* ≤ 0.001. The APD-RPM was associated with a lower mortality rate per patient-year, although this difference was not statistically significant, 0.12 (95% CI: 0.07 to 0.19) versus 0.14 (95% CI: 0.12 to 0.16) events/person-year, *p*=0.468 (Table S1).

## 4. Discussion

The present study in a population of patients with APD, with and without RPM, shows that in a two-year follow-up time horizon, those patients with RPM remained on PD therapy for 3.2 months longer than those without RPM. This result seems of great importance for the patients, although given the observational nature of this study, and the fact that despite the statistical methods used, there is still the possibility of having residual confounding from unmeasured variables (for example, rural/urban living, type of household, and availability of a caregiver), then the results should be taken with caution, and ideally they should be corroborated with a randomized clinical trial. It cannot be excluded that the longer time on therapy among incident APD patients using RPM eventually may be associated with a further increase in the previously reported early dialysis survival advantage of PD compared with in-center HD [17]. In the era of person-centered care, the quality of life that dialysis therapy can provide has become a critical outcome to pursue [18], but it is also evident that the amount of time a patient relies on one type of dialysis treatment is still a core outcome of renal replacement therapies [19] if that therapy is the one chosen following a shared decision-making process [20]. The two most important actions to lengthen the time spent on PD therapy are to decrease the mortality rate and to reduce the technique failure rate. While our study did not show a statistically significant difference in the mortality rate within a two-year follow-up time, it did show a tendency to reduce it in patients with RPM; in this regard, a two-year follow-up time may be too short for this difference to reach statistical significance. Previously, a study by Corzo et al. [16] had already shown that patients using RPM-APD had a significantly lower technique failure rate.

When making comparisons in observational cohorts, a structural difficulty of this type of analysis is that these cohorts are usually unbalanced in terms of predictive variables. To address this challenge, statistical techniques have been developed, such as propensity score matching (PSM), to allow comparisons across balanced subpopulations [21, 22]. The present study used PSM techniques with matching of a substantial number of predictive variables to get two balanced cohorts; we also considered various causes of censorship in this process. In time-to-event studies, time can typically be a biased outcome due to the frequent occurrence of early or late events. With this approach of matching, we could overcome this aspect [6].

Although we did not include data on morbidity or mortality from COVID-19 in the analyses, it should be mentioned that the use of remote monitoring programs for APD patients was very useful during the pandemic to significantly reduce the risk of exposure to the virus when visiting the hospital or care settings, as reported by our group [14].

Some strengths and limitations should be considered when the results of the present study are interpreted. To the best of our knowledge, this is the largest study evaluating the association of APD-RPM vs. APD without RPM with time on therapy. We analysed data from a large network of renal clinics throughout the Colombian geography, with nearly 300 patients in the remote monitoring program, which constitutes a substantial number of participants. Limitations include the observational nature of the study, which precludes conclusions regarding causality. The relatively short follow-up time could result in mortality differences not reaching statistical significance. Although PSM was used successfully to mitigate the impact of the unbalanced patient groups with and without RPM, residual unbalances may persist. Finally, we did not include health-related quality of life measurements and other patient-reported outcome measures, thus leaving out the patient's perspective, which is increasingly recognized to be crucial also for the clinical community and the health care system at large.

## 5. Conclusions

Over two years of follow-up, APD patients supported by RPM stayed 3.2 months longer on APD therapy compared to a matched group of APD patients without RPM. This result indicates that RPM has the potential to improve the clinical effectiveness and the overall quality of APD therapy.

## Figures and Tables

**Table 1:** Baseline characteristics of the full study population according to the exposure status.

Variables	APD-RPM *n* = 288	APD-without RPM *n* = 1176	*p* value
Age, years; mean (SD)	60.2 (16.5)	60.4 (16.1)	0.797
Sex: male, % (n)	63.5 (183)	58.9 (693)	0.152
Diabetes history: yes, % (n)	49.3 (142)	53.5 (629)	0.203
Black race, % (n)	2.4 (7)	7.7 (90)	0.001

*Socioeconomic level, % (n)*			
Low	38.5 (111)	60.3 (709)	<0.001
Medium	57.3 (165)	35.5 (417)	
High	4.2 (12)	4.3 (50)	

*School level, % (n)*			
None	4.9 (14)	13.0 (153)	<0.001
Elementary	29.2 (84)	45.2 (531)	
High school	48.6 (140)	33.7 (396)	
University degree	17.4 (50)	8.2 (96)	

Charlson comorbidity index; mean (SD)	1.9 (1.8)	2.1 (1.8)	0.104
Center size, mean (SD)	137 (43)	118 (49)	<0.001
Hemoglobin, g/dL; mean (SD)	10.4 (1.7)	9.9 (1.7)	<0.001
Phosphorus, mg/dL; mean (SD)	5.4 (1.7)	5.1 (1.6)	0.176
Potassium, mEq/L; mean (SD)	4.9 (0.8)	4.9 (0.8)	0.740
Albumin, g/dL; mean (SD)	3.6 (0.6)	3.5 (0.6)	0.066
Urine output, mL/day; mean (SD)	1006 (695)	971 (674)	0.443
Death events, % (n)	14.9 (43)	19.5 (229)	0.076
Technique failure events,% (n)	16.0 (46)	13.9 (163)	0.359
Kidney transplant events, % (n)	5.2 (15)	2.8 (33)	0.040
Censored events, % (n)	63.9 (184)	63.9 (751)	0.993
Follow-up time, months, mean (SD)	19.0 (7.3)	16.6 (8.0)	<0.001

APD: automated peritoneal dialysis; RPM: remote patient monitoring; SD: standard deviation.

**Table 2:** Mortality cumulative incidence function with analysis of competing risks events in the full sample of APD patients according to exposure status.

Time^*∗*^	APD-RPM *n* = 288			APD-without RPM *n* = 1176		
	CIF	95% CI		CIF	95% CI	
0.5	0.035	0.018	0.062	0.048	0.036	0.061
1	0.071	0.045	0.106	0.110	0.092	0.129
1.5	0.105	0.072	0.145	0.169	0.147	0.193
2	0.159	0.118	0.206	0.236	0.210	0.264

APD: automated peritoneal dialysis; RPM: remote patient monitoring; CIF: cumulative incidence function estimated with the fine & gray model with competing events technique failure and kidney transplant; CI: confidence interval. ^*∗*^Time expressed in years.

**Table 3:** Time on therapy and mortality rate in the full sample and the matched sample.

Outcomes	Full sample			PS matched sample		
	APD-RPM *n* = 288	APD-without RPM *n* = 1176	*p* value	APD-RPM *n* = 287	APD-without RPM *n* = 287	*p* value
Time on therapy,	18.96 (7.32)	16.59 (8.04)	<0.001	18.95 (7.33)	15.75 (8.1)	<0.001
Months, mean (SD)						
Difference, months (95% CI)	2.37 [1.35, 3.39]			3.2 [1.93, 4.46]		

Mortality, events/person-year 95% CI	0.10 [0.07, 0.13]	0.14 [0.12, 0.16]	0.013	0.10 [0.07, 0.13]	0.12 [0.09, 0.16]	0.325
IRR, 95% CI	0.67 [0.47, 0.93]			0.81 [0.54, 1.22]		

SD: standard deviation; RPM: remote patient monitoring; CI: confidence interval; PS: propensity score; IRR: incidence rate ratio defined as APD-RPM/APD-without RPM.

## Data Availability

The data of this study may be requested from the main author: Rafael Mauricio Sanabria; e-mail: mauricio_sanabria@baxter.com.

## Supplementary Materials

Supplementary Materials A supplementary table (S1) is added where the time on therapy estimation is reported after a balancing process of the two cohorts using the inverse probability weighted method (IPTW).
