# Supplementary material for: Time on Therapy of Automated Peritoneal Dialysis with and without Remote Patient Monitoring: A Cohort Study
Source: Int J Nephrol. 2022 Aug 22;2022:8646775. doi: 10.1155/2022/8646775 (PMC9424000; doi:10.1155/2022/8646775)
Supplement: Supplementary Materials — A supplementary table (S1) is added where the time on therapy estimation is reported after a balancing process of the two cohorts using the inverse probability weighted method (IPTW). [file 8646775.f1.docx]

**Table S1. Time on therapy and mortality rate in full sample and weighted sample.**

|  | **Full Sample** | | | **Weighted Sample** | | |
| --- | --- | --- | --- | --- | --- | --- |
| **Outcomes** | **APD-RPM** | **APD-without RPM** | ***p*- value** | **APD-RPM** | **APD-without RPM** | ***p*- value** |
| Time on therapy, | 18.96 (7.32) | 16.59 (8.04) | < 0.001 | 18.99 (4.6) | 16.49 (10.2) | < 0.001 |
| months, mean (SD) |  |  |  |  |  |  |
| Difference, months [95% CI] | 2.37 [1.35, 3.39] | |  | 2.5 [1.16, 3.85] | |  |
| Mortality, events/person-year 95% CI | 0.10 [0.07, 0.13] | 0.14  [0.12, 0.16] | 0.013 | 0.12 [0.07, 0.19] | 0.14  [0.12, 0.16] | 0.468 |
| IRR, 95% CI | 0.67 [0.47, 0.93] | |  | 0.84 [0.53, 1.34] | |  |

SD: Standard deviation; RPM: Remote patient monitoring; CI: Confidence interval; IRR: Incidence rate ratio defined as APD-RPM/APD-Without RPM
